# Ginkgo Biloba L. Residues Partially Replacing Alfalfa Hay Pellet in Pelleted Total Mixed Ration on Growth Performance, Serum Biochemical Parameters, Rumen Fermentation, Immune Function and Meat Quality in Finishing Haimen White Goats

**DOI:** 10.3390/ani11113046

**Published:** 2021-10-25

**Authors:** Yinyin Chen, Xiaoxiao Gong, Tianyu Yang, Maocheng Jiang, Lin Wang, Kang Zhan, Miao Lin, Guoqi Zhao

**Affiliations:** 1 College of Animal Science and Technology, Yangzhou University, Yangzhou 225009, China; chenyinyin90s@163.com (Y.C.); 18762329160@163.com (X.G.); 18762304846@163.com (T.Y.); jmcheng1993@163.com (M.J.); kzhan@yzu.edu.cn (K.Z.); linmiao@yzu.edu.cn (M.L.); 2 Institutes of Agricultural Science and Technology Development, Yangzhou University, Yangzhou 225009, China; michelle861215@hotmail.com; 3 Joint International Research Laboratory of Agriculture and Agri-Product Safety, The Ministry of Education of China, Yangzhou University, Yangzhou 225009, China

**Keywords:** Ginkgo biloba L. residues, pelleted total mixed ration, growth performance, serum biochemical parameters, rumen fermentation, meat quality, Haimen white goats

## Abstract

**Simple Summary:**

This study was conducted to investigate the effects of dietary supplementation with Ginkgo biloba L. residues (GBLR) partially replacing alfalfa hay pellet on growth performance, serum biochemical parameters, rumen fermentation, immune function, and meat quality in finishing Haimen white goats. The results demonstrated that appropriate replacement of alfalfa hay pellet by GBLR in the diets could significantly improve the growth performance, reduce feed cost, and elevate the apparent digestibility of dry matter (DM) and neutral detergent fiber (NDF). Moreover, supplementation with GBLR has potential to enhance the ability of antioxidation, boost immunity, and ameliorate rumen fermentation as well as meat quality. It was concluded that 18% (G18) replacement of alfalfa hay pellet by GBLR was optimal in this experiment.

**Abstract:**

Sixty castrated male Haimen white growing goats with an initial age of 100 days old and similar body weight (16 ± 1.5 kg) were selected and randomly allocated into five groups with three replicates in each group with four goats in each pen (5 m × 3.2 m). Goats in the control group (CG) were fed a basal pelleted total mixed ration supplemented with 30% alfalfa hay pellet, while experimental treatments (G6, G12, G18, or G24) were supplemented with four levels (6%, 12%, 18%, or 24%) of GBLR replacing alfalfa hay pellet in the diet, separately. Results showed that (1) the final body weight, average daily gain, and average feed intake of G18 was significantly higher (*p* < 0.05) than CG; in contrast, the feed conversion ratio of G18 was significantly lower than CG and G12 (*p* < 0.05); the feed cost per head per day of CG was significantly higher (*p* < 0.05) than that of G18 and G24, and exhibited obvious linearly decrease (*p* = 0.04) with increasing GBLR supplementation; and apparent total-tract digestibility of DM and NDF in GBLR treatments were significantly higher (*p* < 0.05) than CG; (2) alanine transaminase (ALT) concentration in the G18 group was significantly lower (*p* < 0.05) than those in the control group; aspartate transaminase (AST) concentration in the G24 was significantly lower (*p* < 0.05) than those in the control group, and an increase in dietary level of GBLR tended to result in a linear decrease (*p* = 0.09) in the concentration of serum AST; (3) the concentration of malondialdehyde (MDA) demonstrated a tendency to decrease (*p* = 0.06) linearly with increasing GBLR supplementation; however, glutathione peroxidase (GSH-PX) activity in G12 was significantly higher (*p* < 0.05) than CG, G6, and G24; in addition, superoxide dismutase (SOD) activity in G18 was significantly higher (*p* < 0.05) than CG and G6; concentration of immunoglobulin M (IgM), immunoglobulin G (IgG), and immunoglobulin A (IgA) were not affected by GBLR, but increasing dietary GBLR showed a tendency (*p* = 0.08) to linearly increase the IgG concentration; the content of interleukin 4 (IL4) was significantly higher (*p* < 0.05) in G12, G18, and G24 than that in CG and G6; (4) There were similar NH_3_-N, pH, TVFA, and butyrate for goats fed different levels of GBLR supplementation; the C2 (*p* = 0.07) and acetate: propionate (*p* = 0.06) demonstrated a tendency to increase linearly with increasing level of GBLR supplementation, separately; however, it was observed that concentration of propionate showed a tendency to decrease (*p* = 0.08) linearly in response to GBLR supplementation; and (5) Increasing dietary GBLR tended to linearly enhance the lightness (L*) (*p* = 0.07) and yellowness (b*) (*p* = 0.09) values of longissimus dorsi muscles; the redness (a*) value in G18 was significantly higher than that in CG (*p* < 0.05).

## 1. Introduction

The healthy breeding of livestock and the production of high-quality animal products require a stable supply of complete feed [1,2,3]. Pelleted total mixed ration (PTMR) is a compound feed, which is a mixture of different feed ingredients and various additives, according to the nutritional needs of ruminants for crude protein, energy, crude fiber, minerals, and vitamins [4,5]. The pelleting process includes a steam conditioning and mechanical pressure, which can partly break down complicated fiber structure and promote starch gelatinization, resulting in increases in feed slaking and nutritional digestibility [6,7,8,9,10]. Compared with the powdered feed, PTMR has been regarded as an efficient feed form for improving the intake, digestibility, feed conversion ratio, and avoiding the ruminant selecting concentrate part, which is regarded as a feasible strategy to raise a fattening ruminant in the intensive feed-lot style feeding system [11,12,13]. However, the sustained high price of imported alfalfa hay pellet and roughage scarcity have become the major limiting factors for fattening goat production in China. Finding and using local alternative feeds have become a feasible and efficient way to offer economic roughage by reducing the feeding costs and mitigating the adverse socio-environmental impacts that would otherwise arise from the disposal of agri-industrial byproducts.

Ginkgo biloba L. (GBL) is from a plant of the ginkgo family that is a Chinese traditional natural medicinal herb and grows in various regions in China. GBL contains more than 20 kinds of flavonoids, terpenoids, phenols, various trace elements, and 17 kinds of amino acids [14]. It has the pharmacological effects of reducing serum cholesterol, scavenging oxygen free radicals, antioxidant, improving cerebral blood circulation, relieving smooth muscle spasm, and relaxing bronchus and bacteriostasis [15,16,17,18,19]. Ginkgo biloba L. residues (GBLR) are the by-products of leaves produced from ginkgo-flavone glycosides and total terpene lactone extraction of GBL. However, most of the GBLR are disposed of by composting, incineration, or in landfill and will ultimately cause environmental contamination. Hence, taking advantage of these byproducts as animal feed might be a possible handling method. Zhou et al. [20] demonstrated that fermented GBLR were valuable protein and fiber sources consisting of 348.6 and 82.6 g/kg, respectively, and dietary supplementation with 10% of fermented GBLR showed the greatest beneficial effects on growth performance, nutrient digestibility, serum biochemical parameters, and immune function in weaned piglets. Previous study has demonstrated that the addition of GBLR affected growth performance, serum biochemistry, and antioxidant capacity of broiler chickens [21]. 

However, a few studies have concentrated on dietary supplementation with GBLR in young ruminants such as lamb and fattening sheep. However, its effect on Haimen white goats have not been reported. Based on the feed value of GBLRs, this study was conducted with the aim to evaluate the effects of GBLR replacing alfalfa hay pellet in PTMR on growth performance, nutrient digestibility, serum biochemical parameters, rumen fermentation, immune function, and meat quality in finishing Haimen white goats to provide some experimental basis for the application of GBLR as ruminant roughage resources in the future.

## 2. Materials and Methods 

### 2.1. Ethical Considerations 

All goats used in this research were strictly cared for in accordance with the principles of Yangzhou University, the Institutional Animal Care and Use Committee (SYXK (Su) IACUC 2016-0019).

### 2.2. Preparation of GBLR and Pelleted Total Mixed Ration

The freshly dried GBLRs were obtained from Xuzhou Ginkgo Source Bio-engineering Co. Ltd in Pizhou city, China. Alfalfa hay pellets were imported from (NAFOSA) Avenida Leizaur, Peralta, Navarra, Spain. GBLRs and alfalfa hay pellets were ground to pass through a 5-mm sieving screen using a cutting mill (Beijing Grinder Instrument Co. Ltd., Beijing, China), and then analyzed for basic nutrient composition (Table 1). All concentrates including ground corn, wheat bran, soybean meal, and DDGS were ground to pass through a 1.2-mm screen. After being totally mixed (SLHSJ7A-Jiangsu Muyang Group Co. Ltd., Yangzhou, China), the diet was pelleted at the 65–75 °C steam temperature with the compression ratio of 9.5:1 to form a cylindrical shape (pellet diameter 6.5 mm; length around 15 mm) using a ring die pellet machine (Jiangsu Muyang Group Co. Ltd., Yangzhou, China).

### 2.3. Experimental Design, Animals, Diets, and Feeding Management

The experiment was conducted at the Ruminant Experiment Research Farm, Xuzhou, China (latitude 33.43° and longitude 116.22°) from March to June in 2019. Sixty castrated male growing Haimen white goats with an initial age at 100 days old, with similar body weight (16 ± 1.5 kg), were selected and randomly allocated into five groups with three replicates in each group with four goats in each pen (5 m × 3.2 m). Goats in the control group (CG) were fed a basal pelleted total mixed ration supplemented with 30% alfalfa hay pellet, while in the experimental treatments (G6, G12, G18, or G24), they were supplemented with four levels (6%, 12%, 18%, or 24%) of GBLRs (dry matter basis) replacing alfalfa hay pellets in the diet, separately. All five different PTMR were adjusted as isonitrogenous and isoenergetic diets (Table 2). The basal diets were formulated to meet or exceed the goats’ nutritional requirements for growing and fattening (Feeding Standard of Meat-producing Sheep and Goats of the People’s Republic of China; NT/T816-2004). During the whole experimental period, all goats were given ad libitum access to PTMR and fresh tap water. All goats were dewormed twice and the goat shed was disinfected before the experiment began. Goats were fed three times at 07:00, 13:00, and 19:00, respectively. Daily feed allocations to each pen were adjusted according to the minimal feed refusals (<10%) in the feed bunk, and the weight of the residual was recorded every day before new feed were delivered. This experiment consisted of a 14-d adaptation and a 70-d fattening period for sample collection.

### 2.4. Sample Collection and Parameter Measurement

During the whole experimental period, all goats were weighed fortnightly before morning feeding, and the average daily gain was calculated. The daily feed offered, orts, and spillages were collected and weighed to determine the average daily feed intake. A 7-day digestion and metabolism test was conducted at the end of the formal period, in which the first two days were the adaptation period and the last five days were sampled. Fecal samples were collected three times a day, with an interval of eight hours for each time. After the collection, 10% H_2_SO4 was added for nitrogen fixation. The 5-day fecal samples (200 g) were mixed and stored in a refrigerator at 20 °C for the determination of the apparent digestibility of nutrients. Feed and feces were dried and ground using a pulverizer for subsequent determination of dry matter, crude protein, neutral detergent fiber, acid detergent fiber, ether extract, and crude ash content. For both PTMR and fecal samples, dry matter was detected using the AOAC method 930.15; the ether extract was measured using the AOAC method 920.85; crude protein was tested using the method described by Kjeldahl with an azotometer (Scino KT260, FOSS, Hillerod, Denmark); neutral detergent fiber and acid detergent fiber were measured using the methods described by Van Soest and a fiber analyzer (2000i, Ankom, New York, NY, USA); and crude ash content was measured according to the AOAC method 938.08.

On the last day of the experiment, rumen fluid (250 mL) was collected through an oral collector and filtered with a four layer sterilized gauze two hours after morning feeding from day 81 to day 84. The pH (PB-21, Beijing Sartorius Scientific Instrument Co. Ltd, Beijing, China) value was determined immediately. Then, 10 mL aliquots were added with 0.1 mL of 6 mol/L HCl and stored at −20 °C for the determination of ammonia nitrogen concentration according to the method of Weatherburn [22]. Another 10 mL of the ruminal fluid samples were thawed in ultrapure water and centrifuged for 10 min at 15,000× *g*. Then, the supernatants were removed to centrifuge tubes containing 25% metaphosphoric acid, then stored at −20 °C for the analysis of volatile fatty acid concentration using a GC ultra gas chromatograph (Thermo Fisher Scientific, Waltham, MA, USA) as described by Guo et al [23]. The yield of crude microbial protein was determined according to the method described by Hall and Herejk [24].

On the last day of the formal period, blood samples were collected before morning feeding. The blood was collected from the jugular vein of goats by vacuum blood sampling, and the blood volume was 10 mL. Blood samples were divided into two parts, 5 mL each, and one was put into the sampling vessel without an anticoagulant. After standing for 30 min, centrifugation was carried out at a rotating speed of 3500 r/min (Eppendorf, Hamburg, Germany) for 15 min. After that, serum was taken and stored in a refrigerator at −80 °C and then used to test for immunoglobulin M (IgM), immunoglobulin G (IgG), immunoglobulin A (IgA), malondialdehyde (MDA), glutathione peroxidase (GSH-PX), superoxide dismutase (SOD), interleukin 1 (IL1), interleukin 6 (IL6), interleukin 2 (IL2), interleukin 4 (IL4), and tumor necrosis factor (TNF-α). Serum samples were sent to the Beijing Sino-UK Institute of Biological Technology, where immunoglobulin IgM, IgG, and IgA concentrations and anti-oxidative indices including SOD, GSH-PX, and MDA activities were determined. Parameters of IL1, IL6, IL2, IL4, and TNF-α were determined referring to the method of Zhan, K et al. [25]. Another blood sample was placed into the blood collection tube containing an anticoagulant, and the total protein, albumin, globulin, creatinine, urea nitrogen, uric acid, glucose, triglycerides, high density lipoprotein, and low density lipoprotein were measured using an automatic biochemical analyzer at the Yangzhou Disease Control and Prevention Center.

At the end of the experiment, four goats were selected from each group for slaughter. The longissimus dorsi (LD) muscle at the 11th to 13th ribs of goats was used to measure the pH, cooking loss, shear force and moisture, crude protein, fat, crude ash, and shear force of muscle, which were determined by referring to the method of Guzman, J.L. and Ripoll, G. et al. [26,27]. The ultimate pH was measured 24 h post-mortem with an Orion 9106 penetrating probe after calibration with two buffers (7.00 and 4.01). Cooking loss was determined 24 h after slaughter by expressing the cooked sample weight (B) as g/100 g of the pre-cooked sample weight (A) as: cooking loss (g/100 g) = [(A − B)/A] × 100. Shear force, expressed as g/100 g of liquid expelled, was determined according to the filter paper press methodology described by American Meat Science Association. Chemical composition of the triceps brachii muscle was determined thrice according to the AOAC standard (1990) methods. The meat color was determined at the same time and location as pH reading. The lightness (L*), redness (a*), and yellowness (b*) values were measured (Chroma meter, CR-400, Tokyo, Japan) for three times on LD for each goat and the average value was used as the result [28].

### 2.5. Statistical Analysis

In this study, all the data were analyzed by one-way analysis of variance (ANOVA) with the PROC general linear model procedure in SAS version 9.2 (Inst. Inc., Cary, NC, USA). The mathematical model used for the analysis was: Yij = μ + Gi + eij where Yij is the responsible variable, μ is the overall mean, Gi is the fixed effect of Ginkgo biloba L. residues (i = 0, 6%, 12%, 18%, or 24% GBLR substitute for alfalfa pellet), and eij is the random residual error. The results were presented with mean values and the standard error of the mean (SEM). Less than 0.05 *p* value was taken to indicate significance and 0.05 < *p* ≤ 0.10 was taken as an indication of tendency.

## 3. Results

### 3.1. Effect of GBLRs Replacing Alfalfa Hay Pellets in PTMR on Growth Performance and Apparent Digestibility of Haimen White Goats

As shown in Table 3, the final body weight, average daily gain. and average feed intake of G18 was significantly higher (*p* < 0.05) than CG. In contrast, the feed conversion ratio of G18 was significantly lower than that of CG and G12 (*p* < 0.05). The feed cost per head per day of CG was significantly higher (*p* < 0.05) than that of G18 and G24, and exhibited obvious linear decrease (*p* = 0.04) with increasing GBLR supplementation. There was no significant difference in other parameters among the groups (*p* > 0.05). Apparent total-tract digestibility of DM and NDF in the GBLR treatments was significantly higher (*p* < 0.05) than CG; however, GBLR had no effect on the apparent total-tract digestibility of EE, ADF, or CP (Table 4).

### 3.2. Effects of GBLRs Replacing Alfalfa Hay Pellets in PTMR on Serum Biochemical Parameters of Haimen White Goats

As presented in Table 5, ALT concentration in the G18 treatment was significantly lower (*p* < 0.05) than those in the control group. AST concentration in G24 was significantly lower (*p* < 0.05) than those in the control group, and an increase in dietary level of GBLR tended to result in a linear (*p* = 0.09) decrease in the concentration of serum AST. ALT concentration in the G18 was significantly lower (*p* < 0.05) than those in the control group. However, concentrations of other serum parameters were not affected by GBLR supplementation.

### 3.3. Effects of GBLRs Replacing Alfalfa Hay Pellets in PTMR on Serum Antioxidants and Immune Parameters of Haimen White Goats

As shown in Table 6, the concentration of MDA demonstrated a tendency to decrease (*p* = 0.06) linearly with increasing GBLR supplementation; however, GSH-PX activity in G12 was significantly higher (*p* < 0.05) than CG, G6, and G24. In addition, SOD activity in G18 was significantly higher (*p* < 0.05) than CG and G6. No significant difference was observed in other groups. As presented in Table 7, concentration of IgM, IgG, and IgA were not affected by GBLR, but increasing dietary GBLR showed a tendency (*p* = 0.08) to linearly increase the IgG concentration. Compared with CG and G6, the content of IL4 was significantly higher (*p* < 0.05) in G12, G18, and G24 than that in CG and G6. However, no differences among treatments were observed in IL1, IL6, IL2, and TNF-α concentrations (*p* > 0.05).

### 3.4. Effects of GBLRs Replacing Alfalfa Hay Pellets in PTMR on Rumen Fermentation of Haimen White Goats

The effects of feeding GBLRs replacing alfalfa pellets in PTMR on fermentation characteristics in the rumen are presented in Table 8. There were similar NH_3_-N, pH, TVFA, and C4 for goats fed different levels of GBLR supplementation in the diet. The C2 (*p* = 0.07) and C2:C3 (*p* = 0.06) demonstrated a tendency to increase linearly with an increase in the level of GBLR supplementation, separately. However, it was observed that the concentration of C3 showed a tendency to decrease (*p* = 0.08) linearly in response to GBLR supplementation. 

### 3.5. Effects of GBLRs Replacing Alfalfa Hay Pellets in PTMR on Meat Quality of Haimen White Goats

As shown in Table 9, the parameters of pH_45min_, shear force, and cooking loss of goat meat were not affected by GBLR treatment (*p* > 0.05). However, increasing dietary GBLR tended to linearly enhance the lightness (L*) (*p* = 0.07) and yellowness (b*) (*p* = 0.09) values of LD muscles. The redness (a*) value in G18 was significantly higher than that in CG (*p* < 0.05). The chemical analysis of goat meat regarding moisture, crude protein, fat, and crude ash did not reveal significant differences in response to GBLR supplementation (*p* > 0.05).

## 4. Discussion

Different types of roughage in the diet would lead to different feed intake and digestibility of the diet, so the growth performance of the animals would therefore be different [29]. Hu et al. [30] reported that ginkgo leaves had no significant effect on the average daily feed intake, final body weight, and average daily gain in sheep. However, our results demonstrated that GBLRs replacing alfalfa hay pellets in PTMR remarkably increased the final body weight, average daily feed intake, and average daily gain while reducing the feeding cost of the goat and the feed conversion ratio, especially in G18. This might be due to the better palatability of GBLRs through extraction to remove ginkgolic acids and the lower price of GBLRs compared with alfalfa hay pellets. Neutral detergent fiber is a common indicator of fiber, derived from the removal of protein, starch, fat, and sugar in feed with neutral detergent, which represents feed volume, determines the satiation of animals, and negatively correlated with feed intake. Its level affects rumen health to a great extent. Hu et al. [30] found that the digestibility of neutral detergent fiber of 15% Ginkgo biloba leaves in supplemental treatment was higher than that of 7.5% Ginkgo biloba leaves supplemental treatment and control group. The result of our research indicate that GBLRs significantly elevated apparent total-tract digestibility of DM and NDF, which was in line with the results of growth performance in the previous study.

Serum biochemical index is an important indicator to reflect the health status and nutrient metabolism function of animals [31]. Alanine transaminase and aspartate transaminase are two important transaminases in the animal body, distributing in tissue cells such as the liver and heart muscle [32]. The activity of both transaminases was significantly increased when the liver was damaged or when protein metabolism was enhanced. Zhang et al. [33] showed that the addition of 0.4% Ginkgo biloba biological feed had no significant difference in ALT and AST compared with the control group, and had no adverse effects on the heart and liver functions of chickens. Our study indicate that an increase in dietary level of GBLRs resulted in a linear decrease in the concentration of serum AST. ALT concentrations in the G18 were significantly lower than those in the CG. However, concentrations of other serum parameters were not affected by GBLR supplementation, which is consistent with the results of previous research. 

Glutathione peroxidase (GSH-PX) is an important peroxidase that exists widely in the body. Its activity can indirectly reflect the ability of free radical scavenging. The higher the level of GSH-PX, the less damage of peroxides to the body. Superoxide dismutase (SOD) is an important component of the antioxidant enzyme system in a biological system. It can catalyze the disproportion of superoxide anion radicals to produce oxygen and hydrogen peroxide, and plays a crucial role in the balance of oxidation and antioxidants in the body. Xu et al. [34] showed that adding 0.25% or 0.50% fermented Ginkgo biloba leaves into the feed could significantly reduce the content of MDA, and significantly increase the activities of SOD and GSH-PX in *Epinephelus*’ liver. In our study, we found that the concentration in MDA demonstrated a tendency to decrease linearly with increasing GBLR supplementation. Additionally, the activities of GSH-PX and SOD were significantly higher in G12 and G18, respectively. Hence, evidence indicates that GBLRs replacing alfalfa hay pellets in the pelleted total mixed ration can improve the antioxidant capability by increasing the activities of antioxidant enzymes in finishing Haimen white goats. The immunoglobulins in animal body are mainly composed of IgA, IgM, and IgG [35]. IgG is produced in the spleen and lymph nodes during humoral immune response, accounting for 70–75% of the total immunoglobulin. It promotes the phagocytosis of mononuclear macrophages, neutralizes the toxicity of bacterial toxins, and combines with viral antigens so that the virus loses its ability to infect host cells, and can directly reflect the immune situation of the animal body [36]. Yang et al. [37] found that 1.0% GBLRs significantly increased the contents of serum globulin and IgG in broilers. Our results are consistent with the previous report and indicate that GBLRs can ameliorate immune performance and have no influence on inflammatory factor parameters.

The internal rumen is a stable anaerobic environment, which is affected by many factors including rumen microorganism, rumen pH, feed type, etc. Rumen fluid pH value is an intuitive indicator to reflect whether the rumen environment is stable. Generally, the rumen pH value fluctuates within the range of 5.5–7.5 [38]. NH_3_-N is an important product of the degradation of nitrogen-containing substances in the rumen such as proteins, peptides, amino acids, and non-protein nitrogen, and is the main source of nitrogen used by rumen microorganisms [39]. Volatile fatty acids (VFAs) are a major energy source for ruminants and could meet 70–80% of total energy requirements for the ruminants. The VFA production in the rumen could be used as an indicator on ration fermentability. The molar proportion of VFA that is yielded could be used to describe whether a ration is appropriate to the livestock [40]. In our study, we found that no differences were observed in rumen pH and NH_3_-N. The data on ruminal short-chain fatty acid measurement showed that C2 and C2:C3 tended to increase linearly with GBLR supplementation while C3 demonstrated a linear decrease with GBLR treatment. These results showed that GBLR treatment had a tendency to improve the ability of rumen fermentation without adverse effects on rumen health.

Physicochemical properties such as pH, cooking loss, water holding capacity, and color are important parameters to evaluate the storage stability of meat [41]. Meat color, which is directly related to its pH, is the first criterion evaluated by consumers and associated with myoglobin and structure of the meat. In general, the lower the cooking loss, the better the juiciness of the meat; the higher the redness (a*), the lower the yellowness (b*) and the lightness (L*), the better the meat color [42]. In this study, we showed that parameters of pH_45min_ and cooking loss of goat meat were not affected by GBLR treatment. However, increasing dietary GBLR tended to linearly enhance the lightness (L*) and yellowness (b*) values of LD muscles. The redness (a*) value in G18 was significantly higher than that in CG. This might be a result of the antioxidant function of GBLR and, thus, a prevention in the oxidation of oxymyoglobin to metmyoglobin. The chemical analysis of goat meat did not reveal significant differences in response to GBLR supplementation in terms of moisture, crude protein, fat, and ash content. These results demonstrate that GBLRs replacing alfalfa hay pellets in PTMR had a tendency to improve the meat quality of Haimen white goats.

## 5. Conclusions

In our study, we found that 18% replacement of alfalfa hay pellets by GBLRs was the optimal level in this experiment. This could significantly improve the feed conversion ratio, reduce the feed cost, elevate the apparent digestibility of DM and NDF, enhance the antioxidant properties, and ameliorate the redness of Haimen white goat meat.

## Figures and Tables

**Table 1:** Nutrient composition of GBLRs and alfalfa hay pellets (DM basis %).

Items	GBLR	Alfalfa Hay Pellet
Dry matter	93.4	93.8
Crude protein	16.5	15.2
Ether extract	1.5	1.6
Crude ash	11.4	11.8
Neutral detergent fiber	40.5	41.2
Acid detergent fiber	32.8	31.0

Abbreviations: DM, dry matter; GBLR, Ginkgo biloba L. residues.

**Table 2:** Ingredients and chemical composition of the diets (DM basis %).

Items	CG	G6	G12	G18	G24
Ground corn	30.5	30.5	30.5	30.5	30.5
Wheat bran	12.2	12.8	13.1	13.2	12.8
Soybean meal (43%)	8.1	8.1	8.4	8.2	8.3
DDGS (corn)	4	3.4	2.8	2.9	3.2
Alfalfa hay pellet	30	24	18	12	6
GBLR	0	6	12	18	24
Peanut hay	10	10	10	10	10
Calcium carbonate	1.0	1.0	1.0	1.0	1.0
Sodium bicarbonate	0.5	0.5	0.5	0.5	0.5
Salt	0.4	0.4	0.4	0.4	0.4
Premix ^1^	3.3	3.3	3.3	3.3	3.3
Nutrient Levels ^2^					
ME (MJ/kg)	9.45	9.46	9.45	9.44	9.45
DM (%)	87.3	88.2	87.5	88.0	87.9
CP (%DM)	14.63	14.52	14.56	14.58	14.60
EE (%DM)	2.34	2.37	2.35	2.38	2.38
Ash (%DM)	9.5	9.6	9.9	9.2	9.4
NDF (%DM)	33.52	32.18	33.84	32.60	31.95
ADF (%DM)	19.30	20.83	20.32	19.90	19.82
Calcium (%DM)	1.15	1.15	1.15	1.15	1.15
Phosphorus (%DM)	0.40	0.40	0.40	0.40	0.40

Abbreviations: CG, control group; DDGS, distiller dried grains with soluble; GBLR, Ginkgo biloba L. residues; ME, metabolic energy; DM, dry matter; CP, crude protein; EE, ether extract; NDF, neutral detergent fiber; ADF, acid detergent fiber; NFE, non-fiber carbohydrates. ^1^ Premix was formulated to provide (per kilogram of the dietary DM) 55 mg of Zn as ZnSO4·7H_2_O; 50 mg of Mn as MnSO_4_·H_2_O; 60 mg of Fe as FeSO_4_·7H_2_O; 25 mg of Cu as CuSO_4_·5H_2_O; 0.5 mg of I as KI; 0.3 mg of Co as CoCl_2_·6H_2_O; 0.5 mg of Se as Na_2_Se·NO_3_; 16,000 IU of vitamin A; 2500 IU of vitamin D and 200 IU of vitamin E. ^2^ Metabolic energy was a calculated value, while the others were measured values; ME = (36.21 × CP + 85.44 × EE + 37.26 × NFE) × 4.184.

**Table 3:** Effect of GBLRs replacing alfalfa hay pellets in PTMR on the growth performance of Haimen white goats.

Items	Treatments					SEM	*p*-Value	
	CG	G6	G12	G18	G24		Linear	Quadratic
Initial BW (kg)	16.1	15.9	16.2	16.0	16.5	0.52	0.35	0.78
Final BW (kg)	28.2 ^b^	29.5 ^a,b^	29.3 ^a,b^	32.1 ^a^	28.9 ^a,b^	0.19	0.63	0.92
ADG (g/d)	193 ^b^	217 ^a,b^	202 ^b^	232 ^a^	205 ^b^	18.2	0.57	0.65
ADFI (kg/d)	1.07 ^b^	1.15 ^a,b^	1.13 ^a,b^	1.20 ^a^	1.12 ^a,b^	0.02	0.16	0.83
FCR	5.60 ^a^	5.30 ^a,b^	5.58 ^a^	5.21 ^b^	5.45 ^a,b^	0.28	0.21	0.33
Feed cost (RMB/head/d)	2.51 ^a^	2.40 ^a,b^	2.37 ^a,b^	2.30 ^b^	2.24 ^b^	0.03	0.04	0.21

Abbreviations: CG, control group; SEM, standard error of the mean; BW, body weight; ADG, average daily gain; ADFI, average daily feed intake; FCR, feed conversion rate. ^a,b^ Values in the same row with different letter superscripts mean significant difference (*p* < 0.05), while with the same or no letter superscripts mean no significant difference (*p* > 0.05), 0.05 < *p* ≤ 0.10 was taken as an indication of tendency.

**Table 4:** Effects of GBLRs replacing alfalfa hay pellets in PTMR on the nutrient apparent digestibility of Haimen white goats.

Items	Treatments					SEM	*p*-Value	
	CG	G6	G12	G18	G24		Linear	Quadratic
DM (%)	0.63 ^b^	0.67 ^a^	0.72 ^a^	0.75 ^a^	0.78 ^a^	0.02	0.15	0.53
EE (%)	0.70	0.71	0.72	0.71	0.70	0.06	0.56	0.98
NDF (%)	0.58 ^b^	0.65 ^a^	0.66 ^a^	0.68 ^a^	0.72 ^a^	0.02	0.22	0.62
ADF (%)	0.54	0.56	0.60	0.62	0.63	0.08	0.40	0.14
CP (%)	0.59	0.65	0.68	0.63	0.66	0.07	0.21	0.58

Abbreviations: DM, dry matter; EE, ether extract; NDF, neutral detergent fiber; ADF, acid detergent fiber; CP, crude protein. ^a,b^ Values in the same row with different letter superscripts mean significant difference (*p* < 0.05), while with the same or no letter superscripts mean no significant difference (*p* > 0.05), 0.05 < *p* ≤ 0.10 was taken as an indication of tendency.

**Table 5:** Effects of GBLRs replacing alfalfa hay pellets in PTMR on the serum biochemical parameters of Haimen white goats.

Items	Treatments					SEM	*p*-Value	
	CG	G6	G12	G18	G24		Linear	Quadratic
TP (g/L)	65.4	66.7	65.9	64.2	67.3	2.36	0.24	0.56
ALB (g/L)	22.6	22.2	21.9	23.1	21.8	0.86	0.19	0.45
GLO (g/L)	44.30	43.97	43.80	42.93	42.38	1.82	0.51	0.68
A/G	0.51	0.52	0.50	0.48	0.49	0.05	0.32	0.79
CREA (μmoI/L)	45.6	50.0	49.5	47.2	46.4	5.43	0.36	0.85
BUN (mmoI/L)	7.12	7.20	7.23	7.22	7.05	0.32	0.13	0.64
UA (μmoI/L)	32.0	33.7	35.2	34.3	31.5	3.42	0.58	0.75
ALT (U/L)	18.2 ^a^	17.5 ^a,b^	17.2 ^a,b^	16.4 ^b^	17.3 ^a,b^	0.81	0.25	0.56
AST (U/L)	62.5 ^a^	59.1 ^a,b^	58.2 ^a,b^	57.5 ^a,b^	56.3 ^b^	0.08	0.09	0.62
GLU (mmoI/L)	4.61	4.59	4.26	4.35	4.20	0.36	0.41	0.86
TG (mmoI/L)	0.21	0.18	0.15	0.13	0.12	0.02	0.24	0.52
HDL (mmoI/L)	0.66	0.62	0.59	0.63	0.61	0.05	0.45	0.72
LDL (mmoI/L)	0.51	0.50	0.55	0.49	0.48	0.06	0.43	0.71

Abbreviations: TP, total protein; ALB, albumin; GLO, globulin; A/G, albumin/globulin; CREA, creatinine; BUN, blood urea nitrogen; UA, uric acid; ALT, alanine transaminase; AST, aspartate transaminase; GLU, Glucose; TG, triglyceride; HDL, high-density lipoprotein; LDL, low-density lipoprotein. ^a,b^ Values in the same row with different letter superscripts mean significant difference (*p* < 0.05), while with the same or no letter superscripts mean no significant difference (*p* > 0.05), 0.05 < *p* ≤ 0.10 was taken as an indication of tendency.

**Table 6:** Effects of GBLRs replacing alfalfa hay pellets in PTMR on the serum antioxidant parameters of Haimen white goats.

Items	Treatments					SEM	*p*-Value	
	CG	G6	G12	G18	G24		Linear	Quadratic
MDA (nmol/mL)	3.46	3.32	3.26	3.22	3.19	0.03	0.06	0.56
GSH-PX (U/mL)	654 ^b^	660 ^b^	684 ^a^	678 ^a,b^	662 ^b^	8.50	0.17	0.72
SOD (U/mL)	75.6^b^	78.5 ^b^	86.3 ^a,b^	91.0 ^a^	87.2 ^a,b^	5.30	0.12	0.41

Abbreviations: MDA, malondialdehyde; GSH-PX, glutathione peroxidase; SOD, superoxide dismutase. ^a,b^ Values in the same row with different letter superscripts mean significant difference (*p* < 0.05), while with the same or no letter superscripts mean no significant difference (*p* > 0.05), 0.05 < *p* ≤ 0.10 was taken as an indication of tendency.

**Table 7:** Effects of GBLRs replacing alfalfa hay pellets in PTMR on the serum immune and inflammatory factors parameters of Haimen white goats.

Items	Treatments					SEM	*p*-Value	
	CG	G6	G12	G18	G24		Linear	Quadratic
lgM (g/L)	0.68	0.67	0.70	0.72	0.71	0.08	0.62	0.81
lgG (g/L)	16.9	17.4	17.5	18.3	18.5	0.12	0.08	0.53
lgA (g/L)	0.64	0.62	0.66	0.67	0.65	0.04	0.27	0.59
IL1 (pg/mL)	30.4	31.4	27.1	28.5	29.4	2.59	0.37	0.65
IL6 (pg/mL)	142	146	139	140	141	12.30	0.43	0.78
IL2 (pg/mL)	317	310	299	305	312	30.20	0.31	0.47
IL4 (pg/mL)	7.13 ^b^	7.28 ^b^	8.15 ^a^	8.17 ^a^	8.32 ^a^	0.35	0.16	0.42
TNF-α (pg/mL)	45.5	49.1	44.3	46.2	49.0	5.20	0.68	0.79

Abbreviations: lgM, immunoglobulin M; lgG, immunoglobulin G; lgA, immunoglobulin A; IL1, interleukin 1; IL6, interleukin 6; IL2,interleukin 2; IL4, interleukin 4; TNF-α, tumor necrosis factor. ^a,b^ Values in the same row with different letter superscripts mean significant difference (*p* < 0.05), while with the same or no letter superscripts mean no significant difference (*p* > 0.05), 0.05 < *p* ≤ 0.10 was taken as an indication of tendency.

**Table 8:** Effects of GBLRs replacing alfalfa hay pellets in PTMR on the rumen fermentation parameters of Haimen white goats.

Items	Treatments					SEM	*p*-Value	
	CG	G6	G12	G18	G24		Linear	Quadratic
NH_3_-N (mg/dL)	8.85	9.10	9.08	9.12	8.89	1.05	0.41	0.83
pH	6.68	6.71	6.80	6.75	6.63	0.58	0.35	0.68
TVFA/(mmol/L)	125.9	123.7	127.6	125.3	126.5	10.82	0.51	0.85
Acetate (C2)/(mmol/L)	56.9	58.0	60.6	62.1	63.5	4.34	0.07	0.42
Propionate (C3)/(mmol /L)	28.8	28.6	27.4	27.2	26.8	2.16	0.08	0.71
Butyrate (C4)/(mmol /L)	7.7	7.6	8.4	7.3	8.1	0.70	0.42	0.78
C2:C3	2.02	2.11	2.20	2.27	2.32	0.14	0.06	0.36

Abbreviations: TVFA, total volatile fatty acids; C2, acetate; C3, propionate; C4, butyrate. Values in the same row with different letter superscripts mean significant difference (*p* < 0.05), while with the same or no letter superscripts mean no significant difference (*p* > 0.05), 0.05 < *p* ≤ 0.10 was taken as an indication of tendency.

**Table 9:** Effects of GBLRs replacing alfalfa hay pellets in PTMR on the meat quality of Haimen white goats.

Items	Treatments					SEM	*p*-Value	
	CG	G6	G12	G18	G24		Linear	Quadratic
pH_45min_	6.42	6.38	6.28	6.19	6.27	0.05	0.28	0.65
Moisture (g/kg)	76.6	76.3	75.5	76.4	75.8	6.65	0.15	0.42
Crude protein (g/kg)	175.8	169.2	171.3	172.5	173.8	15.20	0.26	0.56
Fat (g/kg)	18.5	18.1	17.9	18.3	17.8	2.04	0.35	0.73
Crude ash (g/kg)	13.5	13.2	12.9	13.0	12.8	1.05	0.17	0.46
Cooking loss	26.5	25.8	26.4	25.3	26.2	2.10	0.23	0.72
Shear force (kg/cm^2^)	6.12	6.20	5.98	6.08	6.16	0.04	0.32	0.96
Lightness (L*)	53.20	53.58	53.80	54.01	54.36	4.98	0.07	0.17
Redness (a*)	14.15 ^b^	15.30 ^a,b^	15.40 ^a,b^	15.92 ^a^	15.48 ^a,b^	0.52	0.12	0.52
Yellowness (b*)	6.80	6.89	7.02	7.11	7.20	0.35	0.09	0.15

^a,b^ Values in the same row with different letter superscripts mean significant difference (*p* < 0.05), while with the same or no letter superscripts mean no significant difference (*p* > 0.05), 0.05 < *p* ≤ 0.10 was taken as an indication of tendency.
